# Incidence of Hospitalization due to Influenza‐Associated Severe Acute Respiratory Infection During 2010–2019 in Bangladesh

**DOI:** 10.1111/irv.13352

**Published:** 2024-07-15

**Authors:** Mohammad Abdul Aleem, Katherine Roguski DeBord, Makhdum Ahmed, Mohammed Ziaur Rahman, Mustafizur Rahman, Md Ariful Islam, A. S. M. Alamgir, M. Salimuzzaman, Tahmina Shirin, Mohammod Jobayer Chisti, Mahmudur Rahman, Eduardo Azziz‐Baumgartner, Fahmida Chowdhury, A. Danielle Iuliano

**Affiliations:** ^1^ Program for Emerging Infections, Infectious Diseases Division International Centre for Diarrhoeal Disease Research, Bangladesh (icddr,b) Dhaka Bangladesh; ^2^ National Center for Emerging and Zoonotic Infectious Diseases Centers for Disease Control and Prevention (CDC) Atlanta USA; ^3^ Hematology Oncology AstraZeneca Boston Massachusetts USA; ^4^ One Health Laboratory, Infectious Diseases Division International Centre for Diarrhoeal Disease Research, Bangladesh (icddr,b) Dhaka Bangladesh; ^5^ Virology Laboratory, Infectious Diseases Division International Centre for Diarrhoeal Disease Research, Bangladesh (icddr,b) Dhaka Bangladesh; ^6^ Institute of Epidemiology Disease Control and Research (IEDCR) Dhaka Bangladesh; ^7^ Maternal and Child Nutrition, Nutrition Research Division International Centre for Diarrhoeal Disease Research, Bangladesh (icddr,b) Dhaka Bangladesh; ^8^ Eastern Mediterranean Public Health Network (EMPHNET) Dhaka Bangladesh; ^9^ Global Influenza Branch, Influenza Division Centers for Disease Control and Prevention (CDC) Atlanta Georgia USA; ^10^ Influenza Division Centers for Disease Control and Prevention (CDC) Atlanta USA

**Keywords:** acute respiratory illness, Bangladesh, hospitalization, influenza, respiratory infections, seasonal, surveillance

## Abstract

**Background:**

Global influenza‐associated acute respiratory infections contribute to 3–5 million severe illnesses requiring hospitalization annually, with 90% of hospitalizations occurring among children < 5 years in developing countries. In Bangladesh, the inadequate availability of nationally representative, robust estimates of influenza‐associated hospitalizations limits allocation of resources for prevention and control measures.

**Methods:**

This study used data from the hospital‐based influenza surveillance (HBIS) system in Bangladesh from 2010 to 2019 and healthcare utilization surveys to determine hospital utilization patterns in the catchment area. We estimated annual influenza‐associated hospitalization numbers and rates for all age groups in Bangladesh using WHO methods, adjusted for a 6‐day‐a‐week enrollment schedule, selective testing of specimens from children under five, and healthcare‐seeking behavior, based on the proportion of symptomatic community participants seeking healthcare within the past week. We then estimated national hospitalization rates by multiplying age‐specific hospitalization rates with the corresponding annual national census population.

**Results:**

Annual influenza‐associated hospitalization rates per 100,000 population for all ages ranged from 31 (95% CI: 27–36) in 2011 to 139 (95% CI: 130–149) in 2019. Children < 5 years old had the highest rates of influenza‐associated hospitalization, ranging from 114 (95% CI: 90–138) in 2011 to 529 (95% CI: 481–578) in 2019, followed by adults aged ≥ 65 years with rates ranging from 46 (95% CI: 34–57) in 2012 to 252 (95% CI: 213–292) in 2019. The national hospitalization estimates for all ages during 2010–2019 ranged from 47,891 to 236,380 per year.

**Conclusions:**

The impact of influenza‐associated hospitalizations in Bangladesh may be considerable, particularly for young children and older adults. Targeted interventions, such as influenza vaccination for these age groups, should be prioritized and evaluated.

## 1 Introduction

Globally, influenza‐associated acute respiratory tract infections contribute to 3–5 million severe illnesses requiring hospitalization annually [1]. More than 90% of global influenza hospitalizations among children < 5 years of age occur in developing countries [2], and the number of influenza hospitalizations in Southeast Asia and Africa is three times greater than in other regions [3]. In Bangladesh, almost one‐third of influenza virus infections among children resulted in pneumonia‐associated hospitalizations [4, 5]. Nearly 80% of seasonal influenza‐associated deaths were among persons aged > 60 years, and the influenza‐associated mortality rate among children aged < 5 years was three times greater than that among persons aged 5–60 years [6]. Difficultly accessing healthcare, delays in care‐seeking [7], substandard healthcare services, excess out‐of‐pocket expenses [8], and higher prevalence of comorbid conditions including child malnutrition can result in excess influenza hospitalizations in resource‐limited settings like Bangladesh [9, 10, 11].

However, the lack of nationally representative, robust estimates of influenza‐associated hospitalizations makes it difficult for governments like Bangladesh to effectively prioritize limited resources leading to underutilization of interventions, such as influenza vaccination or antivirals, and contributing to poor pandemic preparedness [12, 13, 14, 15]. There is no national policy or guidelines for influenza vaccines or prophylactic antivirals in Bangladesh. Influenza vaccine is only offered at personal expense in private healthcare sectors, and vaccination coverage is estimated to be less than 1% [16, 17]. One study in Bangladesh showed that only 8% of hospitalized patients with acute respiratory illness received influenza antivirals [18].

The national hospital‐based influenza surveillance (HBIS) system has enrolled World Health Organization (WHO) defined severe acute respiratory illness patients since 2007 and showed the annual circulation of seasonal influenza viruses in Bangladesh typically during May–September [18]. Previous estimates of influenza‐associated hospitalization in Bangladesh were derived from the HBIS system and community‐healthcare utilization survey (HUS) data from four sentinel sites among persons ≥ 5 years (1.1–1.3/100,000 population) during 2008–2010 and children < 5 years (35–60/100,000 population) during 2010–2014 [5, 19]. However, these estimates were generated from only four geographical regions and included just a few annual influenza seasons. Based on HBIS data, influenza virus infection rates have varied annually and across geographical regions within Bangladesh, likely related to different circulating influenza strains. Hence, nationally representative hospitalization estimates should include multiple seasons and geographical locations within the country [18, 20], and periodic updates to estimates would be valuable to evaluate the impact of influenza viruses and changes in population susceptibility to influenza virus infection.

We aimed to update influenza‐associated hospitalization rates in Bangladesh using information from 11 sentinel sites for five age groups during 2010–2019. We applied the WHO *Manual for Estimating Disease Burden Associated with Seasonal Influenza* [21] and extrapolated influenza‐associated hospitalization rates from the 11 sentinel sites to calculate national estimates.

## 2 Methods

### 2.1 Study Sites

**FIGURE 1:**
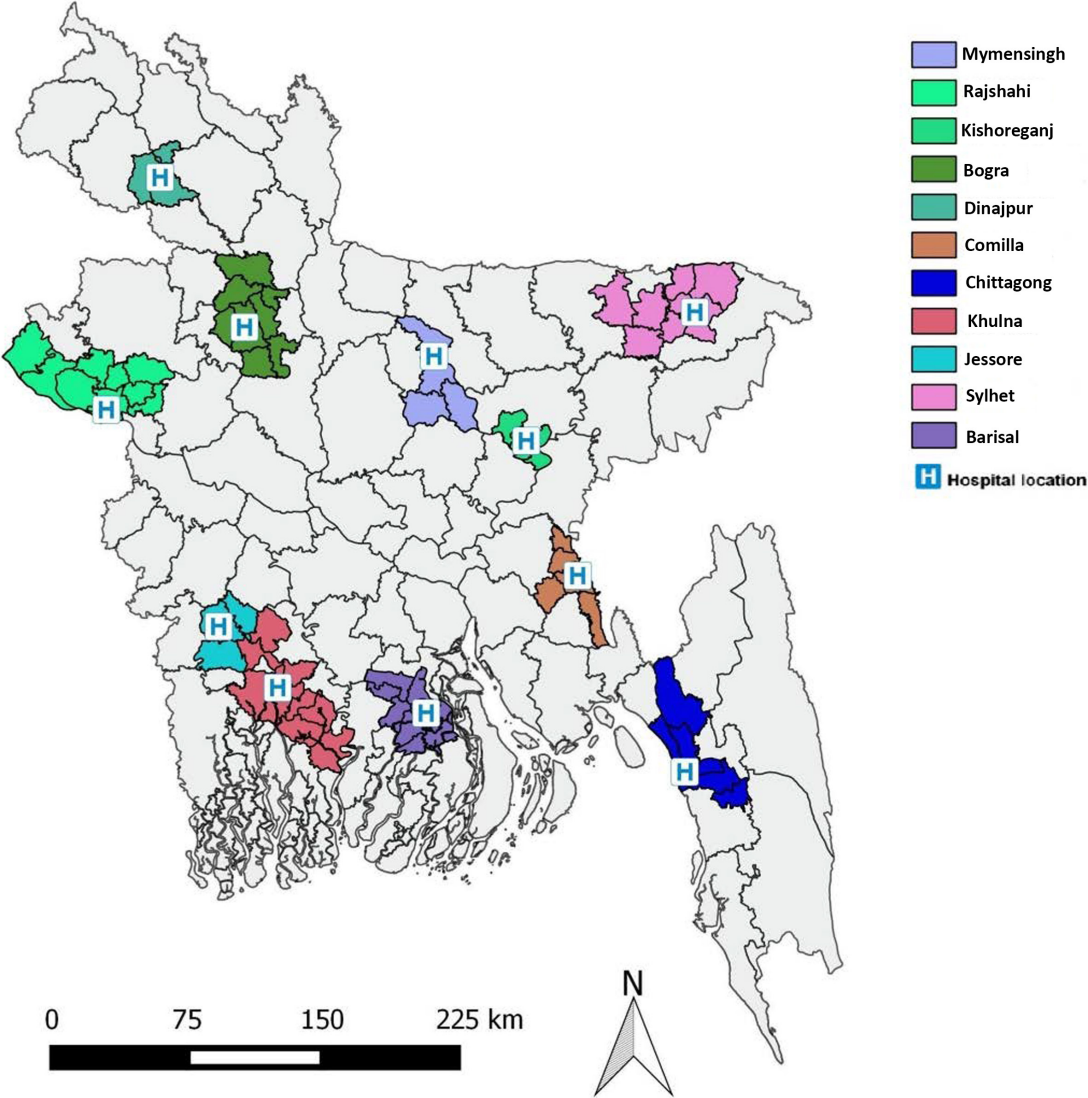
The hospital‐based influenza surveillance hospitals with their catchment areas in Bangladesh. Names of surveillance hospitals with locations: Community Based Medical College Hospital, Mymensingh; Rajshahi Medical College Hospital, Rajshahi; Jahurul Islam Medical College Hospital, Kishoreganj; Shahid Ziaur Rahman Medical College Hospital, Bogra; LAMB Hospital, Dinajpur; Comilla Medical College Hospital, Comilla; BangaBandhu Memorial Hospital, Chittagong; Khulna Medical College Hospital, Khulna; Jessore General Hospital, Jessore; Jalalabad Ragib‐Rabeya Medical College Hospital, Sylhet; Sher‐E‐Bangla Medical College Hospital, Barisal.

We used inpatient surveillance data from the HBIS system [18] from 2010 to 2019 for 11 of the 12 HBIS tertiary care hospital sites (six public and five private) across the eight administrative divisions of Bangladesh. HBIS enrolled patients from the general medicine and pediatric wards throughout the study period and expanded to include intensive care units (ICUs) and cardiac care units (CCUs) in 2018–2019. The locations of the sentinel sites and their catchment areas are illustrated in Figure 1. One HBIS sentinel site, the Dhaka National Medical College Hospital, a private hospital in the capital of Dhaka, was excluded from our analysis because it was not possible to define the hospital's catchment area, as patients may travel to this facility from all over the country. Key demographic and administrative features of the surveillance hospitals are denoted in Table S2. The number of active surveillance sites varied from year to year based on site performance and availability of funding (Table 1 and Table S2).

**TABLE 1:** Methodological changes of hospital‐based influenza surveillance in Bangladesh in chronological order from 2010 to 2019.

Timeline	Changes in case definition
January 2010–June 2016	SARI case definition for persons ≥ 5 years adapted from WHO's updated guideline: An acute respiratory illness with a history of fever OR measured fever > 38°C AND cough OR sore throat, WITH onset of symptom within past 7 days of hospital admission AND requiring hospitalization
	Severe pneumonia (SP) case definition for children < 5 years adapted from WHO's Integrated Management of Childhood Illness (IMCI) guideline: History of cough OR difficulty breathing AND at least one danger sign (i.e., unable to drink or breastfeed, vomits everything, convulsions, lethargy or unconsciousness, chest‐in‐drawing, and stridor in a calm child) WITH onset of symptoms within past 7 days of date of specimen collection AND requiring hospitalization
July 2016–December 2019	WHO's IMCI case definition for SP dropped for children aged < 5 years
	SARI case definition for all ages adapted from WHO's updated guideline [22]: An acute respiratory illness with history of fever OR measured fever ≥ 38°C AND cough, WITH onset of symptoms within past 10 days of date of specimen collection

**Total number of active surveillance sites with defined catchment areas during 2010–2019 included in the current analysis**		
**Timeline**	**Number of sites**	**Surveillance wards**
Jan 2010‐April 2016	11 sites	Medicine and pediatrics
May 2016–Sept 2017	9 sites	Medicine and pediatrics
Oct 2017–Dec 2017	5 sites	Medicine and pediatrics
Jan 2018–Nov 2018	6 sites	Medicine, pediatrics, ICUs, CCUs
Dec 2018–Dec 2019	7 sites	Medicine and pediatrics, ICUs, CCUs

### 2.2 Case Identification, Specimen Collection, and Laboratory Analysis

Detailed descriptions of HBIS and laboratory analysis have been published elsewhere [6, 18, 19, 23]. Briefly, staff enrolled and collected respiratory swabs (nasopharyngeal and throat swabs) from all patients hospitalized with acute respiratory illness meeting the WHO case definitions for severe acute respiratory infection (SARI) [24] and severe pneumonia (SP) [24] 6 days a week. The case definitions of SARI for ages ≥ 5 years [24] and SP for ages < 5 years [24] were used from January 2010 through June 2016 (Table 1). Starting in July 2016, an updated case definition for SARI inpatients for all ages [24] was used. All specimens, collected from patients aged ≥ 5 years, and only the first five specimens collected from children aged < 5 years, were tested per hospital per month. Specimens were tested for seasonal influenza virus A subtypes A (H3N2) and A (H1N1)pdm09, and influenza B, by real‐time reverse transcription polymerase chain reaction (qRT‐PCR) [22]. The number of sentinel sites included in HBIS each year varied from 5 to 11 sites (Table 1).

### 2.3 Hospital Catchment Populations

We defined the catchment areas surrounding the 11 sentinel hospitals (Figure 1) as the unions (lowest administrative units of Bangladesh) where ≥ 75% of the enrolled SARI and SP cases lived. Using previously described methods [5, 23], field staff conducted HUSs in randomly selected unions within catchment areas of each of the 11 surveillance hospitals during July–December 2012 to determine the proportion of the population seeking healthcare at the sentinel hospitals (S) compared to other non‐sentinel hospitals (C) within the catchment area (S/C). Additional details of the HUS methods are in the Supporting Information and published elsewhere [5, 6, 19].

We extracted the age‐specific population of the catchment areas around each sentinel hospital from the 2001 and 2011 national census, reported by the Bangladesh Bureau of Statistics [25, 26]. For 2010, we used an annual growth rate of 1.5% [27] from the 2001 census and for 2011–2019, 1.4% [28] from the 2011 census to estimate the projected catchment populations. Approximately 23,238,000 or 16.3% of the 2011 census population of the country (142,319,000) lived in the 11 sentinel hospital catchment areas. We multiplied the age‐ and sentinel site–specific catchment area census population for every year (P_a_) by the proportion of persons seeking inpatient care at the sentinel hospital compared to all catchment area hospitals derived from the HUS data (S/C) to determine the age‐ and sentinel site–specific catchment populations for every year (D_a_ = P_a_ × [S/C]). We summed the age‐ and sentinel site–specific catchment populations (D_a_) across all 11 sites to determine the total age‐ and year‐specific catchment populations, which we used as the denominators in the national rate calculations (∑D_a_).

### 2.4 Estimation of Age‐Specific Influenza‐Associated Hospitalization Rates

We used the WHO *Manual for Estimating Disease Burden Associated with Seasonal Influenza* [21] to estimate annual influenza‐associated hospitalization rates for all ages and the following age groups: < 5, 5–< 15, 15–< 50, 50–< 65, and ≥ 65 years from 2010 to 2019. Since the number of active surveillance sites varied across months during 2016–2018 (Table 1), we calculated annual estimates during these years using data only from sentinel sites with a full 12 months of surveillance for that year. Only enrolled inpatients residing within the catchment areas were included in the analysis.

We modified the WHO methods to adjust for weekly enrolment practices and low healthcare‐seeking behavior in Bangladesh. We adjusted the monthly number of enrolled case patients (S_a, m_) to account for HBIS enrollment only occurring 6 days a week by multiplying age‐ and site‐specific numbers by the adjustment factor 1.27 (30.4 average days per month/24 days of HBIS enrollment). We also derived an adjustment factor for healthcare‐seeking behavior using the proportion of community participants with fever or cough or difficulty breathing during prior week that sought healthcare at a hospital or clinic out of all community participants reporting one of these symptoms in the prior week.

We then calculated the monthly proportion of specimens positive for influenza viruses (P_m_) as the monthly number of SARI and SP case patients with laboratory‐confirmed influenza divided by the monthly number of SARI and SP case patients tested for influenza viruses across all ages and sentinel hospitals. We calculated an all‐age percent positive because of small numbers of SARI and SP cases enrolled in some age groups in our analyses.

We then estimated age‐specific annual influenza‐associated hospitalization rates using the following formula:

$$R_{a,y}=\frac{\sum_{m=1}^{12}\left(P_{m}\times S_{a,m}\times A_{w}\times A_{\mathrm{hsb}}\right)\times 100,000}{\sum_{s=1}^{11}D_{a,s}}$$

where R_a,y_ is the influenza‐associated hospitalization rates per 100,000 by age group (a) and year (y), P_m_ is the influenza proportion positive across all 11 hospitals for all ages by month (m), S_a,m_ is the total unadjusted number of enrolled SARI or SP case patients across all 11 hospitals by age group (a) and month (m), D_a_ is the catchment population at each of the 11 sentinel hospitals (s) by age group (a) for every year, A_W_ is the adjustment factor for weekly enrollment (1.27), and *A*_hsb_ is the adjustment factor for healthcare‐seeking behavior (5.4).

We then calculated the variance of the number of influenza‐associated annual hospitalizations by age group (Aa,y) using the following formula [29]. We assumed a binomial distribution for the variance in the proportion of samples testing positive for influenza and for the adjustment factor and a Poisson distribution for the variance in SARI hospitalizations:

$$\begin{array}{c} \mathrm{Aa},y=\left[S_{m,a}+{\left(S_{m,a}\right)}^{2}\right]\times \left[\frac{P_{m}\times \left(1-P_{m}\right)}{T_{m}}+{\left(P_{m}\right)}^{2}\right]\\ \;\times \left[\frac{A_{\mathrm{hsb}}\times \left(1-A_{\mathrm{hsb}}\right)}{E}+{A_{\mathrm{hsb}}}^{2}\right]-\left[{\left(P_{m}\right)}^{2}\times {\left(S_{m,a}\right)}^{2}\times {A_{\mathrm{hsb}}}^{2}\right] \end{array}$$

S_m,a_ is the total number of SARI hospitalizations for month (m) and age group (a). P_m_ is the influenza proportion positive across all 11 hospitals for all ages by month (m). *A*_hsb_ is the adjustment factor for healthcare‐seeking behavior. T_m_ is the total number of SARI specimens tested for influenza viruses for month (m). E is the denominator of adjustment factor proportion calculation.

We calculated the 95% confidence intervals (CIs) of age‐specific annual influenza‐associated hospitalizations (H_a,y_) using the following formula:

$$\begin{array}{c} 95\%\;\mathrm{CI}\;\text{of}\;H_{a,y}\;=\;H_{a,y}\;\pm \;\left[1.96\;\times \;\left(\surd A_{a,y}\right)\right] \end{array}$$

The 95% CI of influenza‐associated hospitalization rate (R_a,y_) was calculated using the following formula:

$$\begin{array}{c} 95\%\;\mathrm{CI}\;\text{of}\;R_{a,y}=R_{a,y}\\ \pm \left[1.96\times \left(\surd \left[\left(A_{a,y}\right)÷{\left(\sum_{s=1}^{11}D_{a}\right)}^{2}\right]\times {\left(100,000\right)}^{2}\right)\right] \end{array}$$

### 2.5 National Estimates

We calculated national estimates of total influenza‐associated hospitalization for Bangladesh for all ages, children < 5 years, and older adults aged ≥ 65 years during 2010–2019 by multiplying the age‐specific hospitalization rates calculated from our hospital‐based surveillance data with the age‐specific annual national census population. We calculated the 95% CIs of the national estimates by multiplying the lower and upper bounds of the 95% CIs of the hospitalization rates by the national census population for the corresponding age groups.

### 2.6 Ethics Approval

Enrollment of the participants started after approval of the study by the icddr,b Institutional Review Board (protocol numbers: 2007–002 and PR‐12021). Informed written consent to participate in the study was obtained.

## 3 Results

**FIGURE 2:**
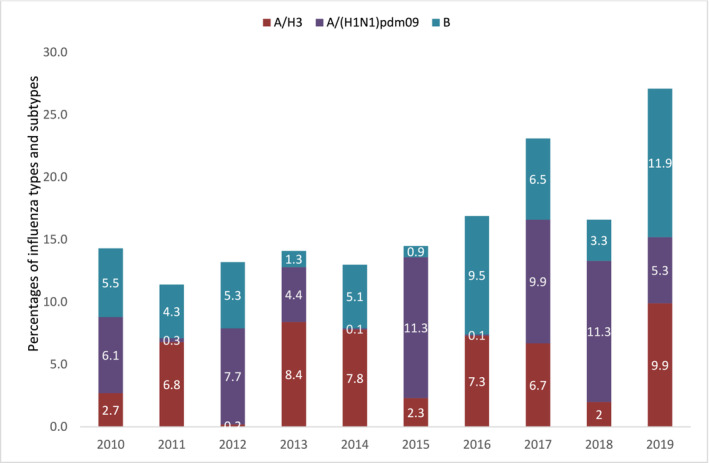
Annual proportions of specimens from severe acute respiratory illness and severe pneumonia patients with laboratory‐confirmed seasonal influenza types and subtypes in Bangladesh, 2010–2019.

During 2010–2019, a total of 23,811 SARI and SP inpatients were enrolled in the 11 study hospitals of the HBIS system (Table S1). Half (12,182, 51.2%) were aged < 5 years, and 15,758 (66.2%) were male (Table S1). Difficulty breathing was the most common symptom (72.4%), followed by a runny nose (52.6%), with chest in‐drawing (85.2%) and abnormal breath sounds (71.1%) prevalent in children under 5, and abnormal chest X‐rays reported in 50.7% of all patients (Table S3). Three‐quarters (17,439, 73.2%) were tested by qRT‐PCR for laboratory evidence of influenza viruses, and among those tested, 3011 (17.3%) had laboratory‐confirmed influenza across 2010–2019 (Table S4). The year with the highest case patient enrollment (2019, *n* = 3621) was also the year with the highest influenza percent positive (27.4%) (Figure 2 and Table S4) and where influenza B was the most frequent circulating virus (11.9%). The lowest annual influenza virus proportion positive was in 2011 (11.5%), where influenza A/H3 contributed to more than half of the positive specimens (6.8%). Higher influenza circulation was typically observed from April through October, peaking in June or July (Figure S1).

### 3.1 Healthcare Utilization

Study staff interviewed 28,846 participants from 6118 households about healthcare utilization. Of these, 915 (3.2%) reported being hospitalized in either sentinel or nonsentinel catchment hospitals during the 12 months preceding the interview (Table S5). Among all persons reporting a hospitalization in the HUS, 243/915 or 27% (95% CI: 23.7%–29.4%) reported hospitalization in sentinel hospitals. The proportion of hospitalizations (sentinel hospitalizations/all catchment hospitalizations) by sentinel site ranged from 1/122 (1%) in Chittagong to 60/96 (62.5%) in Barisal (Table S5). We observed that among 1414 HUS community participants reporting fever or cough or difficulty breathing during the prior week of the interview, 263 (18.6%) sought healthcare at either a catchment hospital or other hospital or qualified medical practitioner, leading to an adjustment factor of 5.4 (100/18.6).

### 3.2 Influenza‐Associated Hospitalizations and Rates

Annual influenza‐associated hospitalization numbers for all ages ranged from 1377 (95% CI: 1178, 1575) in 2011 to 5605 (95% CI: 5222, 5989) in 2019 (Table 2). Almost half and one‐third of all‐age influenza‐associated hospitalizations were among children aged < 5 years and adults aged > 50 years, respectively. Annual influenza‐associated hospitalization rates per 100,000 population across all ages ranged from 31 (95% CI: 27–36) in 2011 to 139 (95% CI: 130–149) in 2019. Influenza‐associated hospitalization rates per 100,000 population, spanning across various calendar years, were highest among children aged < 5 years (range: 114–529) and older adults aged ≥ 65 years (range: 46–252). For children aged < 5 years, influenza hospitalization rates per 100,000 population had three peaks in 2012 (475), 2016 (291), and 2019 (529) when influenza A/(H1N1)pdm09 (7.7%), B (9.5%), and B (11.9%) were most common (Figure 2), respectively, compared to that among other age groups with peaks during 2017 and 2019 when A/(H1N1)pdm09 (9.9%) and B (11.9%) were most common, respectively, among all specimens tested.

**TABLE 2:** Influenza‐associated hospitalization numbers, hospital catchment populations, and influenza‐associated hospitalization rates (per 100,000 population) by age groups and year in Bangladesh, 2010–2019.

Years	Age groups					
	< 5 years	5–< 15 years	15–< 50 years	50–< 65 years	≥ 65 years	All ages
Influenza‐associated hospitalization numbers (95% CI)						
2010	577 (492, 663)	173 (136, 211)	656 (562, 751)	140 (107, 172)	140 (108, 172)	1687 (1479, 1896)
2011	479 (379, 578)	162 (122, 203)	522 (434, 611)	95 (64, 125)	119 (85, 152)	1377 (1178, 1575)
2012	2021 (1755, 2287)	189 (151, 228)	503 (425, 581)	105 (79, 132)	97 (71, 122)	2915 (2553, 3276)
2013	979 (827, 1130)	129 (93, 166)	483 (398, 567)	146 (105, 187)	124 (87, 161)	1861 (1616, 2106)
2014	1048 (887, 1208)	102 (68, 135)	522 (432, 612)	187 (137, 236)	202 (150, 253)	2059 (1801, 2318)
2015	835 (708, 962)	139 (103, 174)	491 (407, 575)	144 (106, 183)	118 (86, 151)	1728 (1496, 1959)
2016	1270 (1100, 1440)	273 (218, 328)	697 (596, 798)	248 (195, 302)	265 (210, 319)	2753 (2448, 3057)
2017	564 (470, 659)	268 (210, 326)	818 (700, 936)	264 (206, 323)	244 (188, 300)	2158 (1913, 2402)
2018	499 (409, 589)	220 (164, 276)	576 (478, 675)	258 (198, 317)	223 (168, 279)	1776 (1558, 1994)
2019	2029 (1843, 2216)	686 (592, 779)	1767 (1602, 1933)	631 (543, 719)	492 (415, 569)	5605 (5222, 5989)
Catchment area populations at risk of hospitalization						
2010	417,322	1,003,093	2,318,636	406,763	217,412	4,363,227
2011	419,580	1,006,130	2,343,830	403,910	209,767	4,383,217
2012	425,328	1,019,914	2,375,941	409,444	212,641	4,443,267
2013	431,155	1,033,887	2,408,491	415,053	215,554	4,504,140
2014	437,062	1,048,051	2,441,487	420,740	218,507	4,565,847
2015	443,050	1,062,410	2,474,936	426,504	221,500	4,628,399
2016	435,910	1,045,864	2,437,910	418,093	218,525	4,556,301
2017	306,851	740,754	1,717,639	298,702	156,824	3,220,770
2018	341,120	822,979	1,895,452	326,851	172,130	3,558,532
2019	383,334	926,497	2,156,220	370,842	195,064	4,031,957
Influenza‐associated hospitalization rates per 100,000 (95% CI)						
2010	138 (118–159)	17 (14–21)	28 (24–32)	34 (26–42)	64 (50–79)	39 (34–43)
2011	114 (90–138)	16 (12–20)	22 (19–26)	23 (16–31)	57 (41–73)	31 (27–36)
2012	475 (413–538)	19 (15–22)	21 (18–24)	26 (19–32)	46 (34–57)	66 (57–74)
2013	227 (192–262)	13 (9–16)	20 (17–24)	35 (25–45)	58 (41–75)	41 (36–47)
2014	240 (203–276)	10 (6–13)	21 (18–25)	44 (33–56)	92 (69–116)	45 (39–51)
2015	188 (160–217)	13 (10–16)	20 (16–23)	34 (25–43)	53 (39–68)	37 (32–42)
2016	291 (252–330)	26 (21–31)	29 (24–33)	59 (47–72)	121 (96–146)	60 (54–67)
2017	184 (153–215)	36 (28–44)	48 (41–54)	88 (69–108)	156 (120–191)	67 (59–75)
2018	146 (120–173)	27 (20–34)	30 (25–36)	79 (61–97)	130 (98–162)	50 (44–56)
2019	529 (481–578)	74 (64–84)	82 (74–90)	170 (146–194)	252 (213–292)	139 (130–149)

### 3.3 National Estimates of Influenza‐Associated Hospitalizations

The annual estimate of influenza‐associated hospitalizations across the entire population of Bangladesh for all ages ranged from 47,891 in 2011 to 236,380 hospitalizations in 2019 (Table 3). The national estimates for children < 5 years ranged from 18,203 to 94,170 and for older adults aged ≥ 65 years from 3517 to 21,436 hospitalizations per year.

**TABLE 3:** National estimates (95% CI) for Bangladesh of influenza‐associated hospitalizations, 2010–2019.

Year	< 5 years	≥ 65 years	All ages
2010	21,589 (18,400–24,660)	3754 (2910–4589)	57,755 (50,741–64,771)
2011	18,203 (14,358–21,854)	4316 (3123–5568)	47,891 (41,180–54,906)
2012	76,833 (66,780–86,878)	3517 (2628–4409)	101,423 (88,128–114,410)
2013	37,213 (31,495–42,908)	4514 (3211–5877)	64,728 (56,910–73,160)
2014	39,834 (33,788–45,980)	7324 (5482–9213)	71,652 (62,961–81,024)
2015	31,754 (26,153–36,937)	4300 (3141–5477)	60,071 (51,536–67,639)
2016	49,778 (42,964–56,290)	9885 (7837–11,923)	98,606 (88,157–109,381)
2017	31,834 (26,512–37,227)	12,867 (9930–15,815)	110,880 (97,640–124,901)
2018	25,672 (21,057–30,358)	10,879 (8220–13,598)	83,712 (73,805–93,945)
2019	94,170 (85,628–102,544)	21,436 (18,106–24,830)	236,380 (221,075–253,187)

## 4 Discussion

We estimated that 48,000–236,000 influenza hospitalizations occur in Bangladesh annually based on 10 years of data from 11 sentinel hospital sites across the country. This estimate represents 3.7%–18% of all estimated hospitalizations in Bangladesh each year [30, 31], raising concerns about the potential strain exerted on the country's healthcare system, economy, and social structure during the study period. Healthcare systems experience overcrowding in hospitals, stretched medical staff, and depleted medical supplies, compromising their ability to address other health issues during the influenza circulation period. Economically, these hospitalizations result in both direct medical costs and indirect costs from lost productivity, particularly affecting families reliant on daily wages, leading to considerable financial stress due to income loss and healthcare expenses. Socially, the burden extends to families and communities, with caregivers missing work to care for sick relatives, amplifying the economic strain and risking broader social disruption in densely populated regions [32]. This comprehensive impact underscores the necessity for strong healthcare strategies in low‐ and middle‐income countries, including prevention and control measures such as vaccination, to effectively manage and control influenza.

Children aged < 5 years had the highest hospitalization rates followed by adults ≥ 65 years and adults 50–< 65 years. Almost half of influenza hospitalizations were among under‐5 children and about one‐third among adults aged ≥ 50 years. Rates among 50–< 65‐year‐old adults were almost double that of younger adults (18–49 years). Our findings underscore influenza's contribution to morbidity among young children and older adults and the need for targeted interventions to lower the risk of influenza virus infections and the associated health burden in Bangladesh. Hospitalization rates varied by age group from 2010 to 2019, possibly because of different circulating influenza virus strains highlighting the importance of routinely updating influenza burden estimates [5, 19].

The current estimates of influenza‐associated hospitalization rates among children < 5 years (114.1–529/100,000 population) are higher than previous reports for Bangladesh: Homaira et al. (20–60/100,000 population) during 2010–2014 [5] and Azziz‐Baumgartner et al. (5.0–22/100,000 population) during 2008–2010 [19]. Our estimates are higher than previous Bangladesh studies, which may be attributed to a longer surveillance period, more surveillance sites, additional age groups, an updated health utilization survey, and use of adjustment factors. In contrast to prior studies, our estimates factored in healthcare‐seeking behavior, significantly influencing the substantial difference observed between our current findings and previous estimates. Healthcare‐seeking behavior in Bangladesh is notably low [28, 33], which may play a crucial role in the discrepancies observed. Our analysis revealed that the adjusted hospitalization numbers were nearly five times higher than unadjusted numbers when health‐seeking behavior was accounted for. This adjustment underpins the marked variance in our estimates, highlighting its critical impact on understanding influenza‐associated hospitalization rates. These methodological changes improve the accuracy of influenza‐associated hospitalization estimates and may be more representative of the Bangladesh population.

Other countries have observed similar burden of influenza‐associated hospitalizations among children and older adults [34, 35]. However, our hospitalization rates for all age groups (31.4–139/100,000 population) were lower than India (44–630/100,000 population) [36]. This difference may be attributed to dissimilarities in surveillance methods, as India enrolled patients from private facilities and included hospitalizations caused by any acute medical exacerbation, beyond those related to respiratory illness. Conversely, our estimates for all ages were higher than tropical low–middle‐income countries in Asia, such as Indonesia (13–19/100,000 population), Thailand (18–111/100,000 population), and Cambodia (9–25/100,000 population) [37]. Our hospitalization rates were similar for children < 5 years, but higher for persons ≥ 5 years compared to countries in Africa, including Kenya [38], Rwanda [39], and Zambia [37]. Several factors may explain the different estimates across countries or regions. One factor is healthcare‐seeking behavior, specifically about one‐third of children with severe respiratory illness globally [40] and in Bangladesh [28] never seek hospital care. Moreover, older adults are less likely to seek care than children in low‐income settings [33]. Shortages and uneven distribution of healthcare resources across geographical regions, out‐of‐pocket expenses, difficulty accessing healthcare, and cultural beliefs are important factors related to lower healthcare seeking in low‐income settings [41, 42]. Aside from differences in healthcare‐seeking behavior, the rate of hospitalization due to influenza may also indicate the virulence of circulating strains in a specific year and factors that make a population more susceptible [34, 37, 38, 39, 43, 44, 45].

## 5 Limitations

We acknowledge important limitations to our influenza‐associated hospitalization estimates. First, patients with symptom onset > 10 days or patients admitted without fever or cough were not eligible for enrollment, which may underestimate the true burden of influenza among hospital admissions. Second, the qRT‐PCR may miss detection of influenza nucleic acid in some cases because of diminished viral shedding, which is sensitive to timing between illness onset and specimen collection [46]. Third, it is possible that some patients may have sought care at district level hospitals or hospitals outside the catchment area and hence not enrolled in the surveillance system. Fourth, we could not adjust for the prevalence of risk factors to acute respiratory infection among catchment population across the sentinel districts. Lastly, we did not test all children < 5 years, which may have introduced bias and led to either over‐ or underestimation of the rate for this age group depending on the actual influenza positivity among the untested children.

## 6 Conclusion

We estimated that 48,000–236,000 influenza‐associated hospitalizations occur in Bangladesh every year. Our analysis provided updated estimates of influenza‐associated hospitalization burden in Bangladesh based on a greater number of study sites, a longer surveillance period, and adjustments for catchment healthcare‐seeking behavior. The influenza hospitalization burden was higher among children less than 5 years of age, older adults, and elderly compared to other age groups in Bangladesh. Policies around control of influenza prioritizing the influenza vaccination among these high‐risk groups may help reduce the burden of influenza‐associated hospitalizations in Bangladesh.

## Author Contributions

**Mohammad Abdul Aleem:** writing–original draft, conceptualization, methodology, formal analysis, project administration, supervision, funding acquisition, data curation. **Katherine Roguski DeBord:** conceptualization, writing–review and editing, methodology, formal analysis, resources. **Makhdum Ahmed:** conceptualization, methodology, writing–review and editing, formal analysis, project administration. **Mohammed Ziaur Rahman:** methodology, writing–review and editing, formal analysis, project administration, resources. **Mustafizur Rahman:** methodology, writing–review and editing, formal analysis, project administration, resources. **Md Ariful Islam:** writing–review and editing, formal analysis, project administration, data curation. **A.S.M. Alamgir:** methodology, project administration, resources. **M. Salimuzzaman:** methodology, project administration, resources. **Tahmina Shirin:** writing–review and editing, project administration, resources, supervision. **Mohammod Jobayer Chisti:** writing–review and editing. **Mahmudur Rahman:** writing–review and editing, project administration, resources, supervision. **Eduardo Azziz‐Baumgartner:** conceptualization, writing–review and editing, formal analysis, funding acquisition. **Fahmida Chowdhury:** writing–review and editing, project administration, formal analysis, supervision. **A. Danielle Iuliano:** conceptualization, methodology, writing–review and editing, formal analysis, supervision.

## Conflicts of Interest

The authors declare no conflicts of interest.

### Peer Review

The peer review history for this article is available at https://www.webofscience.com/api/gateway/wos/peer‐review/10.1111/irv.13352.

## Supporting information


**Table S1.** Sociodemographic characteristics of patients with severe acute respiratory illness and severe pneumonia in Bangladesh, 2010–2019.
**Table S2.** Demographic, healthcare, and administrative features of the influenza surveillance hospitals in Bangladesh, 2010–2019.
**Table S3.** Clinical characteristics of patients with severe acute respiratory illness and severe pneumonia in Bangladesh, 2010–2019.
**Table S4.** Annual proportions of laboratory‐confirmed seasonal influenza types and subtypes among patients with severe respiratory illness and severe pneumonia in Bangladesh, 2010–2019.
**Table S5.** History of hospitalizations among community participants in 11 catchment areas during 12 months preceding the healthcare utilization survey, July–December 2012.

**Figure S1** Annual influenza seasonality among severe acute respiratory illness and severe pneumonia patients in Bangladesh, 2010–2019.

**Data S1** Supporting Information.

## Data Availability

Data generated during the study are subject to a data access policy of icddr,b and are available from icddr,b’s research administration on reasonable request through the corresponding author.
