# Supplementary material for: Incidence of Hospitalization due to Influenza‐Associated Severe Acute Respiratory Infection During 2010–2019 in Bangladesh
Source: Influenza Other Respir Viruses. 2024 Jul 15;18(7):e13352. doi: 10.1111/irv.13352 (PMC11247272; doi:10.1111/irv.13352)
Supplement: Supplementary file 1 — Table S1. Sociodemographic characteristics of patients with severe acute respiratory illness and severe pneumonia in Bangladesh, 2010–2019. Table S2. Demographic, healthcare, and administrative features of the influenza surveillance hospitals in Bangladesh, 2010–2019. Table S3. Clinical characteristics of patients with severe acute respiratory illness and severe pneumonia in Bangladesh, 2010–2019. Table S4. Annual proportions of laboratory‐confirmed seasonal influenza types and subtypes among patients with severe respiratory illness and severe pneumonia in Bangladesh, 2010–2019. Table S5. History of hospitalizations among community participants in 11 catchment areas during 12 months preceding the healthcare utilization survey, July–December 2012. [file IRV-18-e13352-s001.docx]

Supplementary Table 1: **Socio-demographic characteristics of patients with severe acute respiratory illness and severe pneumonia in Bangladesh, 2010-2019**

| **Years** | **2010**  **N=1,721**  **n (%)** | **2011 N=2,128**  **n (%)** | **2012 N=3,090**  **n (%)** | **2013 N=2,160**  **n (%)** | **2014 N=2,663**  **n (%)** | **2015 N=1,950**  **n (%)** | **2016 N=2,659**  **n (%)** | **2017**  **N=2,222 n (%)** | **2018**  **N=1,597 n(%)** | **2019**  **N=3,621 n(%)** | **All years**  **N=23,811**  **n (%)** |
| --- | --- | --- | --- | --- | --- | --- | --- | --- | --- | --- | --- |
| **Age** | | | | | | | | |  |  |  |
| < 5 years | 593 (34.5) | 1,072 (50.4) | 2,114 (68.4) | 1,382 (64.0) | 1,680 (63.1) | 1,071 (54.9) | 1,423 (53.5) | 832 (37.4) | 565 (35.4) | 1,502 (41.5) | 12,182 (51.2%) |
| 5 – 14 years | 158 (9.2) | 180 (8.5) | 174 (5.6) | 124 (5.7) | 110 (4.1) | 149 (7.6) | 242 (9.1) | 240 (10.8) | 144 (9.0) | 376 (10.4) | 1,949 (8.2%) |
| 15 – 49 years | 666 (38.7) | 598 (28.1) | 551 (17.8) | 422 (19.5) | 497 (18.7) | 444 (22.8) | 597 (22.5) | 694 (34.2) | 429 (26.9) | 1,034 (28.6) | 5,932 (24.9%) |
| 50 – 64 years | 149 (8.7) | 122 (5.7) | 128 (4.1) | 126 (5.8) | 175 (6.6) | 137 (7.0) | 176 (6.6) | 244 (11.0) | 259 (16.2) | 392 (10.8) | 1,908 (8.0%) |
| ≥65 years | 155 (9.0) | 156 (7.3) | 123 (4.0) | 106 (4.9) | 201 (7.5) | 149 (7.6) | 221 (8.3) | 212 (9.5) | 200 (12.5) | 317 (8.8) | 1,840 (7.7%) |
| **Male** | 1,125 (65.4) | 1,441 (67.7) | 2,060 (66.7) | 1,455 (67.4) | 1,809 (67.9) | 1,314 (67.4) | 1,787 (67.2) | 1,457 (65.6) | 1,059 (66.3) | 2,251 (62.2) | 15,758 (66.2) |

Supplementary Table 2. **Demographic, healthcare and administrative features of the influenza surveillance hospitals in Bangladesh, 2010- 2019**

| **Sl.** | **Districts** | **Location** | **Administration** | **Catchment population served** | **Initiation** | **Activities as of December 2019** | **No. of**  **beds*** | **No. of non-sentinel health facilities in the catchment area** |
| --- | --- | --- | --- | --- | --- | --- | --- | --- |
| 1 | Mymensingh | Rural | Private | 2,055,655 | October 2007 | Ended in October 2017 | 600 | 33 |
| 2 | Kishoregonj | Rural | Private | 430,967 | September 2007 | Ongoing | 400 | 2 |
| 3 | Rajshahi | Urban | Public | 4,083,067 | May 2007 | Ongoing | 650 | 39 |
| 4 | Bogra | Urban | Public | 845,373 | September 2007 | Ongoing | 500 | 30 |
| 5 | Dinajpur | Rural | Private | 922,431 | October 2007 | Ended in May 2016 | 500 | 17 |
| 6 | Chittagong | Urban | Private | 4,726,700 | August 2007 | Ended in May 2016 | 250 | 37 |
| 7 | Comilla | Urban | Public | 959,383 | October 2007 | Ongoing | 250 | 38 |
| 8 | Khulna | Urban | Public | 4,044,821 | July 2007 | Ongoing | 250 | 39 |
| 9 | Jessore | Urban | Public | 1,447,315 | September 2007 | Ongoing | 300 | 12 |
| 10 | Sylhet | Urban | Private | 3,194,561 | July 2007 | Ongoing | 750 | 29 |
| 11 | Barisal | Urban | Public | 527,545 | August 2007 | Ongoing | 500 | 18 |

*Average bed occupancy is 148.21% [28]

Supplementary Table 3: **Clinical characteristics of patients with severe acute respiratory illness and severe pneumonia in Bangladesh, 2010-2019**

| **Clinical features** | **2010**  **n (%)** | **2011**  **n (%)** | **2012**  **n (%)** | **2013**  **n (%)** | **2014**  **n (%)** | **2015**  **n (%)** | **2016**  **n (%)** | **2017**  **n (%)** | **2018**  **n (%)** | **2019**  **n (%)** | **Total**  **n (%)** |
| --- | --- | --- | --- | --- | --- | --- | --- | --- | --- | --- | --- |
|  | **All age patients** | | | | | | | | | | |
|  | **N=1,721** | **N=2,128** | **N=3,090** | **N=2,160** | **N=2,661** | **N=1,950** | **N=2,659** | **N=**  **2,222** | **N=**  **1,597** | **N=**  **3,621** | **N= 23,809** |
| Running nose | 971 (56.4) | 1,251 (58.8) | 1,846 (59.7) | 1,218 (56.4) | 1,283 (48.2) | 904 (46.4) | 1,173 (44.1) | 1,116 (50.2) | 695 (43.5) | 2,066 (57.1) | 12523 (52.6) |
| Diarrhea | 37 (2.1) | 50 (2.3) | 69 (2.2) | 42 (1.9) | 44 (1.7) | 27 (1.4) | 58 (2.2) | 71 (3.2) | 19 (1.2) | 75 (2.1) | 492 (2.1) |
| Difficulty breathing | 1,289 (74.9) | 1,647 (77.4) | 2,542 (82.3) | 1,730 (80.1) | 2,165 (81.4) | 1,534 (78.7) | 1,836 (69.0) | 1,265 (56.9) | 978 (61.2) | 2,260 (62.4) | 17,246 (72.4) |
| Abnormal breath sounds | 891(51.8) | 902 (55.2) | 836 (53.7) | 1,037 (59.6) | 1,845 (69.5) | 1,242(63.7) | 1,548 (58.5) | 1,123 (50.5) | 776 (48.6) | 2,113 (58.4) | 12,313 (57.7) |
|  | **N=1,139** | **N=1,073** | **N=992** | **N=798** | **N=998** | **N=893** | **N=1,265** | **N= 1,426** | **N= 1,048** | **N= 2,119** | **N=**  **1,1751** |
| Sore throat* | 321 (28.2) | 310 (28.9) | 225 (22.7) | 159 (19.9) | 83 (8.3) | 64 (7.2) | 166 (13.4) | 195 (13.7) | 210 (20) | 646 (30.5) | 2,381 (20.2) |
| Headache* | 557 (48.9) | 611 (56.9) | 556 (56) | 390 (48.9) | 368 (36.9) | 344 (38.4) | 705 (55.7) | 883 (61.9) | 515 (49.1) | 1,239 (58.5) | 6,168 (52.5) |
| Chills* | 53 (4.7) | 62 (5.8) | 81 (8.2) | 70 (8.8) | 22 (2.2) | 23 (2.6) | 47 (3.7) | 49 (3.4) | 22 (2.1) | 38 (1.8) | 467 (4) |
| Body ache* | 189 (16.6) | 317 (29.5) | 372 (37.5) | 280 (35.1) | 282 (28.3) | 274 (30.7) | 543 (42.9) | 702 (49.2) | 476 (45.4) | 1,156 (54.6) | 4,591 (39.1) |
|  | **N=637** | **N=826** | **N=1,304** | **N=1,043** | **N=1,195** | **N=886** | **N=1,059** | **N=910** | **N=776** | **N= 1,786** | **N= 1,0422** |
| Abnormal chest X-ray*** | 294 (46.2) | 431 (52.2) | 717 (55.0) | 526 (50.4) | 762 (63.8) | 487 (55.0) | 503 (47.5) | 362 (39.8) | 364 (46.9) | 842 (47.1) | 5,288 (50.7) |
|  | - | - | - | **N=1,165** | **N=2,008** | **N=47** | **N=63** | **N=75** | **N=105** | **N=216** | **N= 3,679** |
| At least one Chronic illness**** | - | - | - | 146 (12.5) | 382 (19) | 47 (100) | 63 (100) | 75 (100) | 105 (100) | 216 (100) | 1,034 (28.1) |
| **Patients aged <5 years** | | | | | | | | | | | |
|  | **N=582** | **N=1,055** | **N=2,098** | **N=1,262** | **N=1,664** | **N=1,057** | **N=1,394** | **N=796** | **N=546** | **N= 1,502** | **N= 1,1956** |
| Chest indrawing | 515 (88.5) | 1,003 (95.1) | 1,993 (95.0) | 1,112 (88.1) | 1,608 (96.6) | 933 (88.3) | 1,121 (80.4) | 553 (69.5) | 370 (67.8) | 975 (64.9) | 10,183 (85.2) |
| Stridor in a calm child | 106 (18.2) | 197 (18.7) | 397 (18.9) | 214 (17.4) | 120 (7.2) | 94 (8.9) | 101 (7.2) | 32 (4) | 71 (13) | 101 (6.7) | 1,433 (12) |
| Being unable to drink | 99 (17.0) | 223 (21.1) | 400 (19.1) | 265 (21.2) | 339 (20.4) | 392 (37.1) | 471 (33.8) | 178 (22.4) | 137 (25.1) | 363 (24.2) | 2,867 (24) |
| Lethargy or unconsciousness | 43 (7.4) | 127 (12.0) | 196 (9.3) | 141 (11.3) | 89 (5.3) | 71 (6.7) | 50 (3.6) | 16 (2) | 6 (1.1) | 20 (1.3) | 759 (8.4) |
| Vomits everything | 108 (18.6) | 150 (14.2) | 290 (13.8) | 191 (15.3) | 262 (15.7) | 202 (19.1) | 228 (16.4) | 165 (20.7) | 70 (12.8) | 170 (11.3) | 1,836 (15.4) |
| History of convulsions | 31 (5.3) | 92 (8.7) | 97 (4.6) | 91 (7.3) | 65 (3.9) | 57 (5.4) | 94 (6 .7) | 65 (8.2) | 40 (7.3) | 101 (6.7) | 733 (6.1) |
|  | **N=582** | **N=561** | **N=564** | **N=941** | **N=1,657** | **N=1,056** | **N=1,387** | **N=796** | **N=549** | **N= 1,500** | **N= 9,593** |
| Abnormal breath sounds | 404 (69.4) | 394 (70.2) | 397 (70.4) | 676 (71.8) | 1,259 (76.0) | 756 (71.6) | 910 (65.6) | 535 (67.2) | 328 (59.7) | 1,163 (77.5) | 6,822 (71.1) |
|  | **N=582** | **N=1,050** | **N=2,097** | **N=1,354** | **N=1,656** | **N=1,057** | **N=1,387** | **N=796** | **N=549** | **N= 1,502** | **N= 1,2030** |
| Abnormal chest X-ray *** | 96 (16.5) | 249 (23.7) | 511 (24.4) | 321 (23.7) | 433 (26.1) | 273 (25.8) | 242 (17.4) | 134 (16.8) | 152 (27.7) | 397 (26.4) | 2,808 (23.3) |

* Questions are asked to participants aged ≥5 years only;

**Questions are asked to participants aged >18 years;

***Not all X-rays are done;

****Chronic illness includes asthma, COPD, diabetes, hypertension, ischemic heart disease, cancer, malaria, kidney disease, liver disease, AIDS; for patients aged ≥5 years

Supplementary Table 4: **Annual proportions of laboratory-confirmed seasonal influenza types and subtypes among patients with severe respiratory illness and severe pneumonia in Bangladesh, 2010-2019.**

| **Years** | **Total specimens tested** | **Total influenza positives*** | **A (H3N2)**** | **A(H1N1)pdm09**** | **Influenza B**** |
| --- | --- | --- | --- | --- | --- |
| 2010 | 1721 | 246 (14.3%) | 47 (19.1%) | 105 (42.7%) | 94 (38.2%) |
| 2011 | 1668 | 191 (11.5%) | 113 (59.2%) | 5 (2.6%) | 72 (37.7%) |
| 2012 | 1722 | 228 (13.2%) | 4 (1.8%) | 133 (58.8%) | 91 (39.9%) |
| 2013 | 1482 | 209 (14.1%) | 124 (59.3%) | 65 (31.1%) | 19 (9.1%) |
| 2014 | 1722 | 225 (13.1%) | 134 (60.4%) | 2 (0.9%) | 88 (39.1%) |
| 2015 | 1384 | 201 (14.5%) | 32 (15.9%) | 156 (77.6%) | 12 (6.0%) |
| 2016 | 1743 | 296 (17.0%) | 128 (43.2%) | 1 (0.3%) | 166 (56.1%) |
| 2017 | 1803 | 417 (23.1%) | 121 (29.0%) | 178 (42.7%) | 117 (28.1%) |
| 2018 | 1403 | 233 (16.6%) | 28 (12.0%) | 158 (67.8%) | 47 (20.2%) |
| 2019 | 2791 | 765 (27.4%) | 275 (35.9%) | 148 (19.3%) | 333 (45.5%) |
| All years | 17439 | 3011 (17.3%) | 1006 (33.5%) | 951 (31.6%) | 1039 (34.0%) |

*Total number of influenza positives was divided by total number tested to derive overall percent proportion.

** Number of influenza strain positives was divided by total influenza positives to derive strain specific percent proportion

Supplementary Table 5. **History of hospitalizations among community participants in 11 catchment areas during 12 months preceding the health care utilization survey, July-December 2012**

| **Districts** | **Admissions at**  **all hospitals** | **Admissions**  **at sentinel hospitals, n (%)** | **Admissions at**  **non-sentinel catchment hospitals, n (%)** |
| --- | --- | --- | --- |
| Mymensingh | 76 | 12 (16) | 64 (84) |
| Kishoreganj | 53 | 26 (49) | 27 (51) |
| Rajshahi, | 96 | 30 (31) | 66 (69) |
| Bogra | 99 | 37 (37) | 62 (63) |
| Dinajpur | 21 | 2 (10) | 19 (90) |
| Chittagong | 122 | 1 (1) | 121 (99) |
| Comilla | 115 | 32 (28) | 83 (72) |
| Khulna | 127 | 25 (20) | 102 (80) |
| Jessore | 38 | 10 (26) | 28 (74) |
| Sylhet | 72 | 8 (11) | 64 (89) |
| Barisal | 96 | 60 (63) | 36 (38) |
| **All districts** | **915** | **243 (27)** | **672 (73)** |
