# Supplementary material for: Incidence of Hospitalization due to Influenza‐Associated Severe Acute Respiratory Infection During 2010–2019 in Bangladesh
Source: Influenza Other Respir Viruses. 2024 Jul 15;18(7):e13352. doi: 10.1111/irv.13352 (PMC11247272; doi:10.1111/irv.13352)

Supplementary Figure 1: **Annual influenza seasonality among severe acute respiratory illness and severe pneumonia patients in Bangladesh, 2010-2019.**

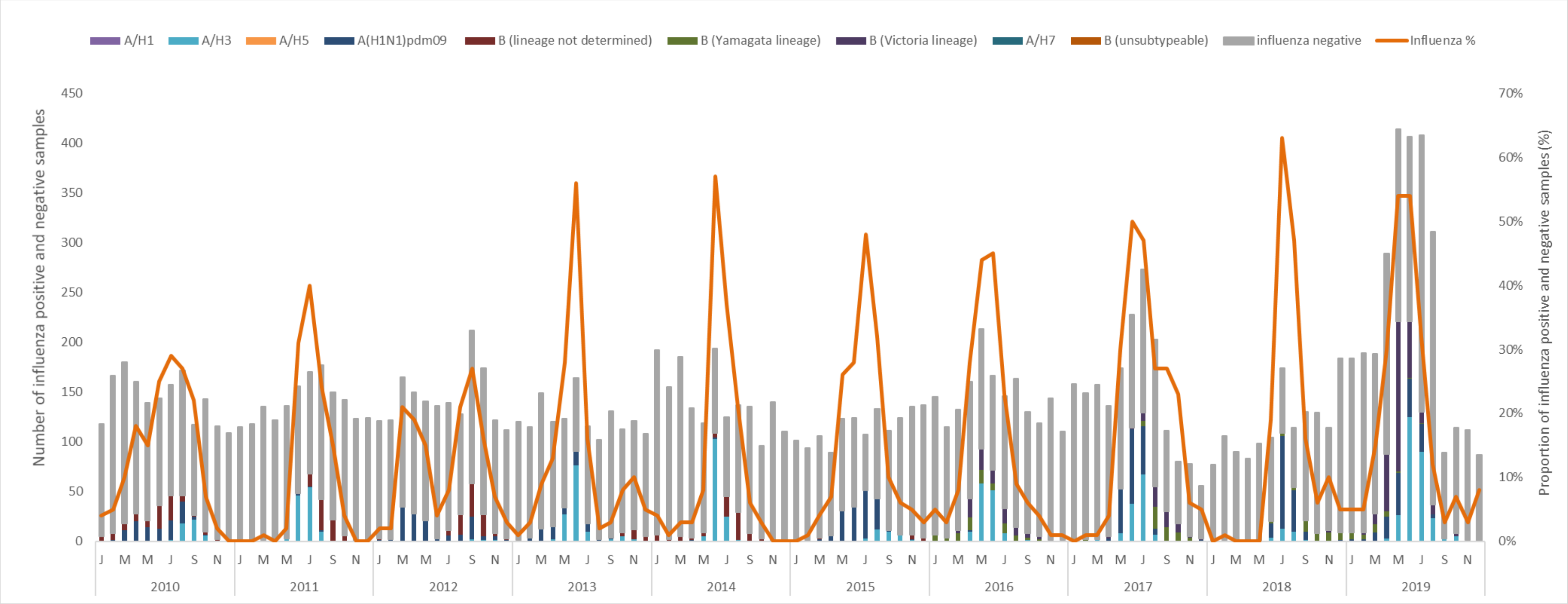

Supplement: Supplementary file 2 — Figure S1 Annual influenza seasonality among severe acute respiratory illness and severe pneumonia patients in Bangladesh, 2010–2019. [file IRV-18-e13352-s002.pdf]
