# Supplementary material for: Incidence of Hospitalization due to Influenza‐Associated Severe Acute Respiratory Infection During 2010–2019 in Bangladesh
Source: Influenza Other Respir Viruses. 2024 Jul 15;18(7):e13352. doi: 10.1111/irv.13352 (PMC11247272; doi:10.1111/irv.13352)
Supplement: Supplementary file 3 — Data S1 Supporting Information. [file IRV-18-e13352-s003.docx]

**SUPPLEMENTARY DOCUMENT**

**Title: Incidence of hospitalization due to influenza-associated severe acute respiratory infection during 2010-2019 in Bangladesh**

**Socio-demographic characteristics of patients with severe acute respiratory illness and severe pneumonia in Bangladesh, 2010-2019:**

The *Supplementary Table 1* shows the socio-demographic characteristics of patients with severe acute respiratory illness and severe pneumonia in Bangladesh from 2010-2019. The table shows the number and percentage of patients by age group and gender for each year. The data shows that patients aged under 5 years had the highest proportion of severe respiratory illness and pneumonia cases over the years, with a peak of 68.4% in 2012. Males accounted for the majority of cases each year, with a range of 62.2% to 67.9%. The proportion of cases among adults aged 50 years and above increased gradually over the years, with the highest proportion in 2019. The data highlights the vulnerable groups and can help inform targeted interventions for prevention and control of severe respiratory illness and pneumonia in Bangladesh.

**Demographic, healthcare and administrative features of the influenza surveillance hospitals in Bangladesh, 2010- 2019:**

The *Supplementary Table 2* provides information about the demographic, healthcare, and administrative features of the influenza surveillance hospitals in Bangladesh from 2010 to 2019. The table lists the 11 sentinel hospital sites across the country, their location, administration, catchment population served, initiation, and activities as of December 2019, the number of beds and non-sentinel health facilities in the catchment area. The average bed occupancy is also mentioned, which is very high (148.21%). It shows that most of the surveillance hospitals are located in urban areas and are administered either by public or private entities. The hospitals have been operational since 2007, and most are ongoing, except for a few that ended during the surveillance period. We can see that out of the 11 surveillance hospitals in Bangladesh, 5 are administered by the public sector and 6 by the private sector. Therefore, the ratio of public to private hospitals in this particular surveillance network is approximately 1:1.2. The initiation of the activities of the surveillance hospitals in Bangladesh varied between May 2007 and October 2007. Most of the hospitals (8 out of 11) started their activities in 2007, while two hospitals started in 2008, and one in 2010. This suggests that the surveillance system was gradually established over time, and it took a few years to have a sufficient number of hospitals to cover different regions of the country. District 3 (Rajshahi) has the highest number of non-sentinel health facilities in the catchment area (39), while district 2 (Kishoregonj) has the lowest (2). The hospitals in Rajshahi and Khulna serve populations of over 4 million, while the hospital in Kishoreganj serves a much smaller population of around 430,000. This suggests that the distribution of healthcare resources is not uniform across the country, with some areas having access to more healthcare facilities than others. This could have implications for the ability of the healthcare system to respond to public health crises, such as an outbreak of a contagious disease. The total catchment population served by private administration is 11,330,314 and the total catchment population served by public administration is 11,908,504. Therefore, the total catchment population served by public administration is slightly higher than that served by private administration.

**Clinical characteristics of patient with severe acute respiratory illness and severe pneumonia:**

The *Supplementary Table 3* presents clinical features of patients with severe acute respiratory illness and severe pneumonia in Bangladesh from 2010 to 2019. The table shows the number and percentage of patients with different symptoms such as running nose, diarrhea, difficulty breathing, abnormal breath sounds, sore throat, headache, chills, body ache, abnormal chest X-ray findings, and pre-existing chronic illness. The data are presented for all age patients, patients aged <5 years, and separately for different years.

According to the table, difficulty breathing was the most common symptom among all age patients, reported by 72.4% of enrolled patients. Running nose was also common, reported by more than half of the patients (52.6%). Among children aged <5 years, chest in-drawing was the most common symptom (85.2%), followed by abnormal breath sounds (71.1%). Abnormal chest X-ray findings were reported in almost half of the enrolled patients (50.7%).

The table also shows that the prevalence of some symptoms varied across different years. For example, the percentage of patients with sore throat and headache increased over the years, while the percentage of patients with chills and body ache decreased. Additionally, the table indicates that pre-existing chronic illness was more common in later years, with 28.1% of enrolled patients in 2019 reporting at least one chronic illness.

**Annual proportions of laboratory-confirmed seasonal influenza types and subtypes among patients with severe respiratory illness and severe pneumonia in Bangladesh, 2010-2019:**

The *Supplementary Table 4* presents data on the number of specimens tested for influenza and the number of influenza positives, broken down by the subtypes A(H3N2), A(H1N1)pdm09, and Influenza B, for each year from 2010 to 2019. The total number of specimens tested increased from 1,721 in 2010 to 2,791 in 2019. The total number of influenza positives also increased from 246 in 2010 to 765 in 2019, with an overall positivity rate of 17.3%. The percentage of positive specimens for influenza varied from 11.5% in 2011 to 27.4% in 2019. The highest percentage of positives for A(H3N2) was in 2017 (29.0%), while the highest percentage for A(H1N1)pdm09 was in 2018 (67.8%). Influenza B had the highest percentage of positives in 2016 (56.1%). Overall, Influenza B had the highest percentage of positives among the subtypes, followed by A(H1N1)pdm09 and A(H3N2). The data suggest that there is variability in the prevalence of different influenza subtypes over time.

**Influenza seasonality:**

Higher influenza circulation was typically observed during April or May through October with peak influenza activity in June or July most of the calendar years (*Supplementary Figure 1*). In year 2012, bimodal influenza peaks were observed during May-April (predominant A(H1N1)pdm09) and August-September (predominant influenza B). Year-round influenza activities were observed during 2012, 2013, 2016 and 2018. Across all seasons the influenza proportion positive during April-October was 22.8% and during June-July, 37.1%. The highest influenza proportion ranged from 21% in March 2012 to 60% in July 2018.

**Healthcare Utilization Survey (HUS):**

Within the catchment area for each sentinel hospital, the number of all-cause hospitalizations at each sentinel hospital (S) across all ages was added to all-cause hospitalizations at non-sentinel hospitals (O) across all ages to calculate the total number of all-cause hospitalizations in the catchment area (C= S+O). The number of sentinel hospitalizations (S) was then divided by the total number of hospitalizations within the catchment area across all ages (C) to determine the proportion of persons hospitalized at sentinel hospitals (S/C) for all causes and across all ages. To calculate the sentinel site-specific hospital utilization rate in the catchment area, we used site-specific all-cause hospitalizations, because the number of respiratory hospitalizations was too small to produce reliable information. Also, we used number of hospitalizations across all ages instead of age-specific hospitalization because the number of age-specific hospitalization was also small, potentially introducing bias. This approached allowed us to improve the reliability of our sentinel hospital-utilization rate estimation.

**History of hospitalizations among community participants in 11 catchment areas during 12 months preceding the health care utilization survey, July-December 2012:**

The *Supplementary Table 5* presents data on hospital admissions in various districts of Bangladesh. The table includes three columns: admissions at all hospitals, admissions at sentinel hospitals (expressed as a percentage), and admissions at non-sentinel catchment hospitals (also expressed as a percentage).

The data shows that the total number of admissions at all hospitals is 915. Among them, 243 (27%) admissions occurred at sentinel hospitals, and 672 (73%) admissions occurred at non-sentinel catchment hospitals. It is noteworthy that Barisal has the highest number of hospital admissions (96), with 63% (60) of admissions occurring at sentinel hospitals and 38% (36) of admissions occurring at non-sentinel catchment hospitals.

The data also indicates that the percentage of admissions at sentinel hospitals varies widely across districts, ranging from 1% in Chittagong to 63% in Barisal. Overall, the percentage of admissions at sentinel hospitals is 27% across all districts.

In summary, the data shows that the percentage of hospital admissions at sentinel hospitals is relatively low across all districts of Bangladesh, with wide variation across districts. The majority of hospital admissions occur at non-sentinel catchment hospitals.
